# Metastatic Non–Small Cell Lung Cancer Mimicking Metaplastic Breast Cancer: A Case Report

**DOI:** 10.1200/PO.24.00027

**Published:** 2024-06-21

**Authors:** Tanmayi S. Pai, Blake McKinley, Robert Seby, Jason T. Lewis, Miglena K. Komforti, Joseph Accurso, Sushilkumar Sonavane, Deborah A. Baumgarten, Pooja P. Advani, Yanyan Lou, Rohit Rao

**Affiliations:** ^1^ Mayo Clinic, Jacksonville, FL

## Abstract

NGS used to diagnose and treat NSCLC patient with initial concern for metaplastic breast cancer

## Introduction

Metastasis of non–small cell lung cancer (NSCLC) to the breast is rare, with only four reports of this phenomenon in female patients to our knowledge,^1^ but it is critical to distinguish it from a primary breast malignancy. Treatment of these two malignancies can be drastically different, particularly in an era where targeted therapies and immunotherapy have revolutionized the management of metastatic NSCLC (mNSCLC). Extended immunohistochemistry (IHC) and next-generation sequencing (NGS) can aid diagnosis in cases of undifferentiated breast malignancy. Commonly used IHC markers for lung cancer include TTF-1, napsin-A, CK5/6, CK7, p40, CK20, and p63. Conversely, IHC markers for breast cancer (BC) include GATA3, GCDFP-15, TRPS1, estrogen receptor (ER), progesterone receptor (PR), and human epidermal growth factor receptor 2 (HER2). Here, we present a unique case of an elderly woman with a symptomatic breast mass initially diagnosed as a primary breast malignancy. On extended IHC evaluation and tissue NGS via FoundationOne CDx's platform, she was found to have mNSCLC with a driver mutation. Although the disease initially responded well to targeted therapy, she soon developed oligoprogressive disease. Resistance mutations to targeted therapy were detected on tissue and peripheral blood (PB) NGS, assayed using Tempus's xT CDx and Guardant360's platforms, respectively.

## Case Presentation

A 74-year-old African American woman presented to the oncology clinic complaining of a palpable left breast mass that had grown rapidly over 1 month, fatigue, night sweats, and 60-pound unintentional weight loss over 1 year. Her medical history included hypertension and a 10-pack-year smoking history. Her family history was notable for her sister's diagnosis of BC at age 40 years. On physical examination, a 4-centimeter left breast mass was palpated. Lymph node examination was unremarkable.

**FIG 1.**
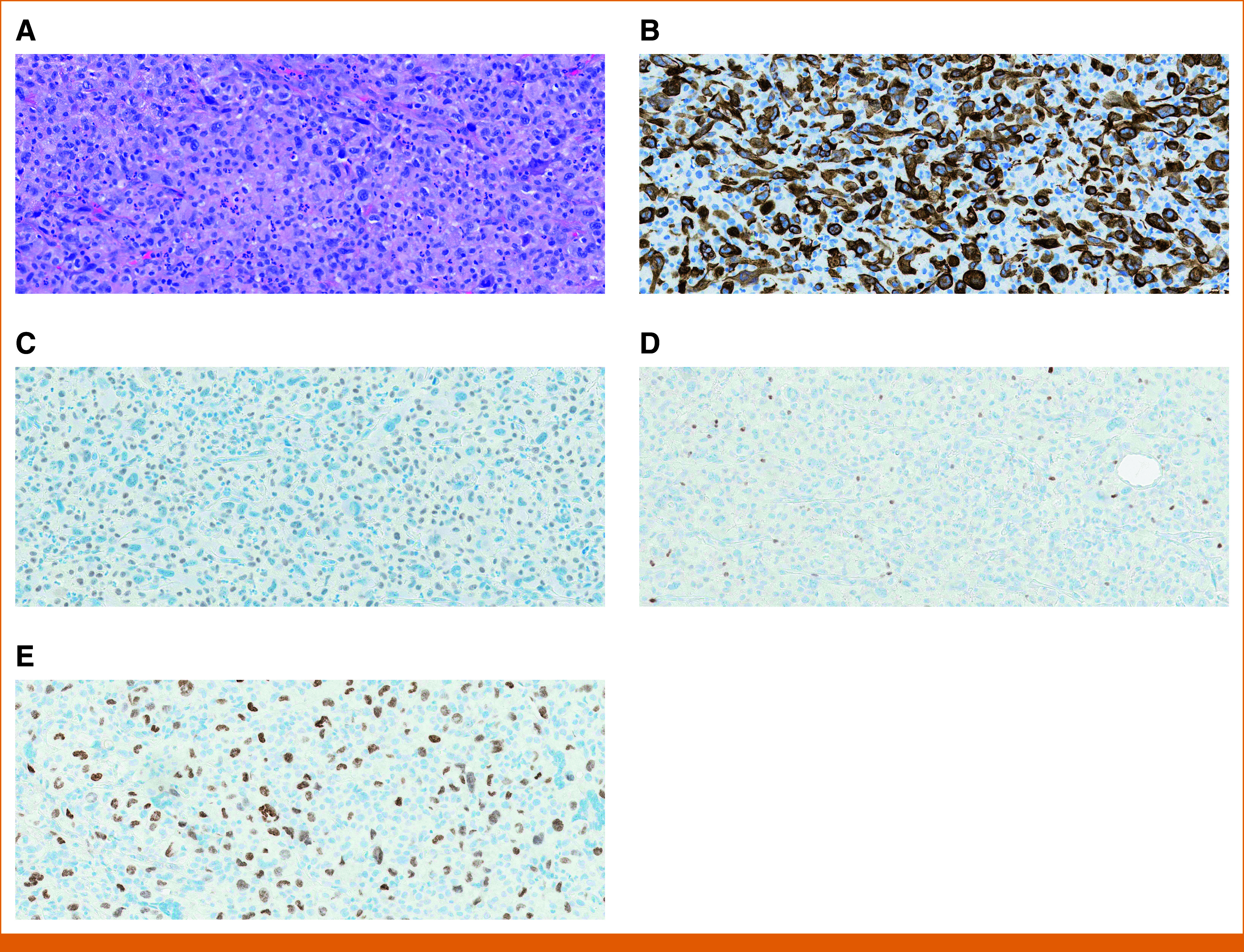
(A) Breast mass, needle biopsy: H&E stain demonstrating high-grade, poorly differentiated tumor. (B) Strongly positive cytokeratin AE1/AE3 immunohistochemical stain supports epithelial origin. (C) Breast mass, needle biopsy: TRPS1 IHC stain showing nonspecific low-level nuclear staining with weak intensity. (D) Negative GATA3 immunohistochemical stain. (E) Positive TTF-1 immunohistochemical stain. Of note (slides not shown), p63, CK5, ER, PR, and Melan-A IHC stains were also negative. HER2 IHC was indeterminate because of cytoplasmic expression; HER2 FISH was negative. ER, estrogen receptor; FISH, fluorescence in situ hybridization; H&E, hematoxylin and eosin; HER2, human epidermal growth factor receptor 2; IHC, immunohistochemistry; PR, progesterone receptor.

Diagnostic mammogram revealed a 3.6-cm irregular, circumscribed mass in the left breast's upper outer quadrant, with ultrasound of the mass showing heterogeneity and hypervascularity with multiple thick intervening septations. Ultrasound-guided biopsy of the mass revealed high-grade poorly differentiated carcinoma (Fig 1A). On IHC evaluation, tumor cells were strongly positive for epithelial markers CAM5.2 and keratin AE1/AE3; weakly positive for BC marker TRPS1; and negative for BC markers GATA3, ER, PR, and HER2-neu; squamous cell carcinoma markers p63 and CK5; and melanoma marker SOX10 (Figs 1B-1D). Given the inconclusive IHC evaluation, triple-negative metaplastic breast cancer (MBC), a heterogeneous entity that can encompass varying cytomorphologies, was tentatively diagnosed.

**FIG 2.**
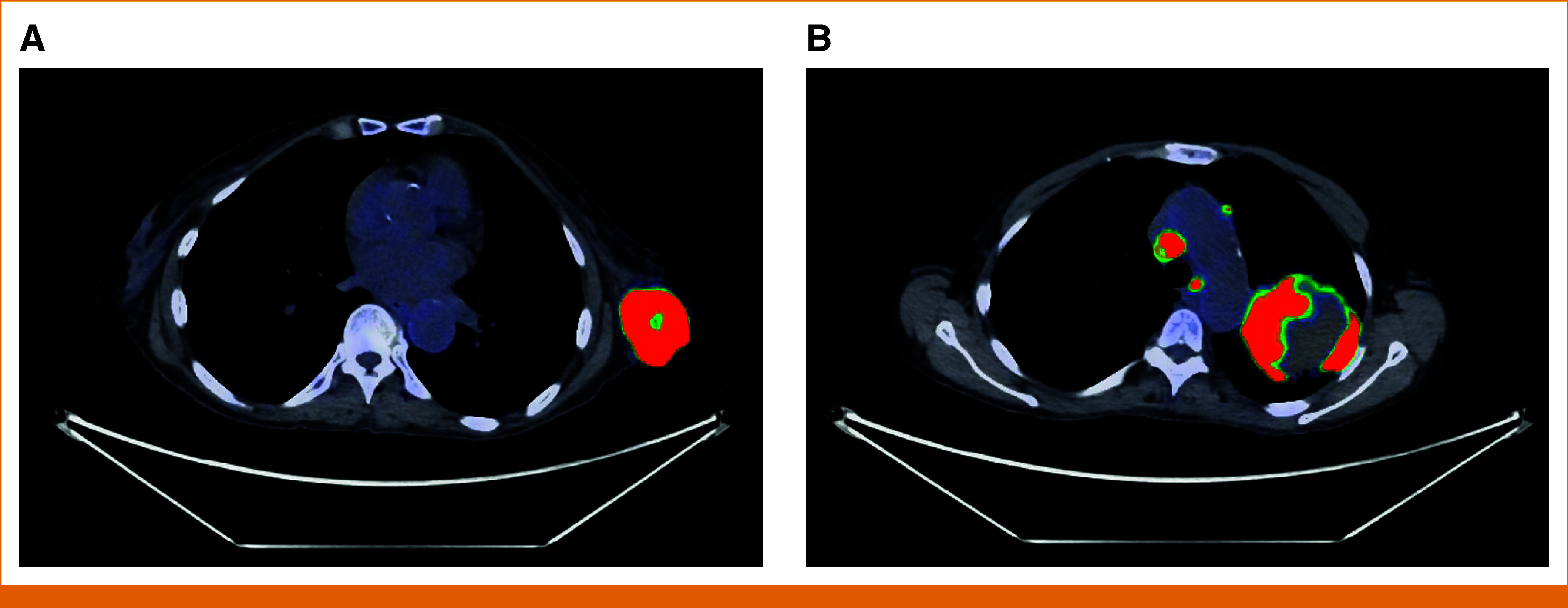
(A) Intensely hypermetabolic left breast mass approximately 4.5 cm in diameter, maximum SUV 26.4, on FDG PET-CT. (B) Greater than 7 cm intensely hypermetabolic left lung consolidation, maximum SUV 33.3. Hypermetabolic right supraclavicular, mediastinal, and right axillary/subpectoral lymphadenopathy was also noted on PET-CT, with a maximum SUV of 28.9 involving right supraclavicular lymphadenopathy. Additional hypermetabolic subcentimeter pulmonary nodules, the largest subpleural measuring 0.7 cm in the right lower lobe with a maximum SUV of 6, and 0.5-cm pericardial nodule with mild hypermetabolism, with a maximum SUV of 3.5, were also visualized. FDG PET-CT, fluorodeoxyglucose positron emission tomography-computed tomography; SUV, standardized uptake value.

However, concern remained that the mass reflected metastasis from a nonbreast primary tumor because of the patient's constitutional symptoms, which suggested a systemic rather than a localized disease process. Fluorodeoxyglucose positron emission tomography-computed tomography (FDG PET-CT) was ordered. Imaging demonstrated an intensely hypermetabolic left lung consolidation, pulmonary nodules, and extensive thoracic lymphadenopathy (Fig 2).

**FIG 3.**
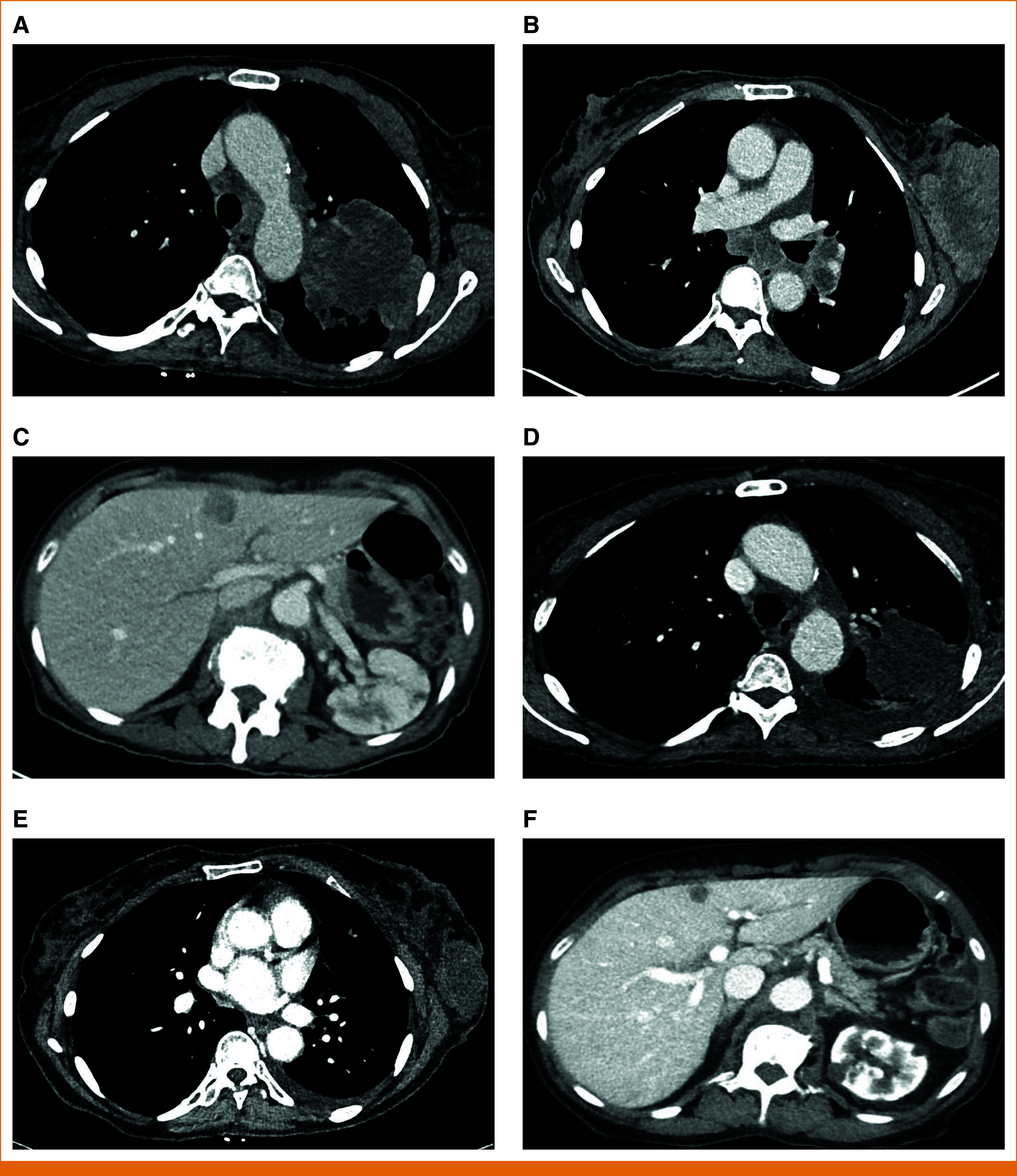
(A) Axial image from chest CT with contrast shows a lobulated heterogeneously enhancing mass in the left upper lobe measuring up to 7.9 cm. (B) Necrotic subcarinal, left perihilar lymphadenopathy (arrows). A large lobulated heterogeneous left breast mass measuring up to 9.4 cm (partially imaged) is also noted. (C) CT of abdomen with contrast performed before treatment shows 22 × 20 mm left hepatic mass. (D) Axial image from chest CT with contrast performed 3 months after treatment shows the interval decrease in size of left upper lobe mass and mediastinal and left perihilar lymphadenopathy. (E) Axial image from chest and left breast mass CT with contrast performed 3 months after treatment with increased areas of low attenuation within. (F) CT of abdomen with contrast performed 3 months after treatment shows the interval decrease in size of hepatic metastatic lesion to 11 × 12 mm. CT, computed tomography.

As suspicion rose for a primary lung tumor, additional IHC staining on the original breast biopsy was requested. Tissue NGS was sent simultaneously and a mesenchymal-epithelial transition (MET) exon 14 skipping c.28818-18_2888-4del mutation (NM_000245.2) was identified, suggesting that the breast mass was metastatic from a lung primary (Table 1). Tumor cells were positive for TTF-1, consistent with metastatic lung adenocarcinoma (Fig 1E). Subsequent full NSCLC staging revealed significant interval enlargement of the breast mass, the known lung findings, liver metastasis (Figs 3A-3C), and unremarkable brain imaging. The patient was deemed eligible for treatment with capmatinib, a tyrosine kinase inhibitor (TKI) targeted therapy for mNSCLC with MET exon 14 skipping mutation.

**TABLE 1.** Next-Generation Sequencing at Diagnosis and Disease Progression

Timing of NGS Assessment	Platform	Specimen Source	Molecular Alteration (HGVS nomenclature)	Variant Allele Frequency (tissue)/Cell-Free DNA Amplification (blood), %
Initial diagnosis (pretreatment)	FoundationOne CDx	Breast tissue	MET c.2888-18_2888-4del (NM_000245.2)	21.9
			CDKN2A/B p16INK4a D74Y and p14ARF R88L (NM_000077.4)	15.1
			TP53 p.R273L (NM_000546.4)	15.5
Progression of disease (6 months post-treatment)	Guardant360	Peripheral blood	MET c.2888-18_2888-4del (NM_000245.2)	0.7
			MET p.Y1230C (NM_000245.4(MET):c.3689A>G (p.Tyr1230Cys))	0.3
			MET p.Y1230H (NM_000245.4(MET):c.3688T>C (p.Tyr1230His))	0.1
			TP53 p.Y220H (NM_000546.6(TP53):c.658T>C (p.Tyr220His))	0.1
			TP53 p.R273L (NM_000546.6(TP53):c.818G>T (p.Arg273Leu))	0.5
	Tempus xT CDx	Adrenal gland tissue	MET c.2888-18_2888-4del (NM_000245)	9.7
			MET p.Y1230H (NM_000245.4(MET):c.3688T>C (p.Tyr1230His))	9.3
			TP53 p.R273L (NM_000546.6(TP53):c.818G>T (p.Arg273Leu))	14.4
			CDKN2A p.D74Y (NM_000077)	12.3

NOTE. HGVS nomenclature was obtained from FoundationOne CDx and ClinVar (accessed March 1, 2024).

Abbreviations: HGVS, human genome variation society; NGS, next-generation sequencing.

The patient completed a course of palliative external beam radiation therapy (20 Gy) to the left breast and started twice-daily capmatinib 400 mg. She tolerated the treatment well, with mild peripheral edema as the only drug-related adverse effect. Restaging scans obtained 3 months after treatment initiation demonstrated near-partial treatment response with a marked decrease in disease burden (Figs 3D-3F).

**FIG 4.**
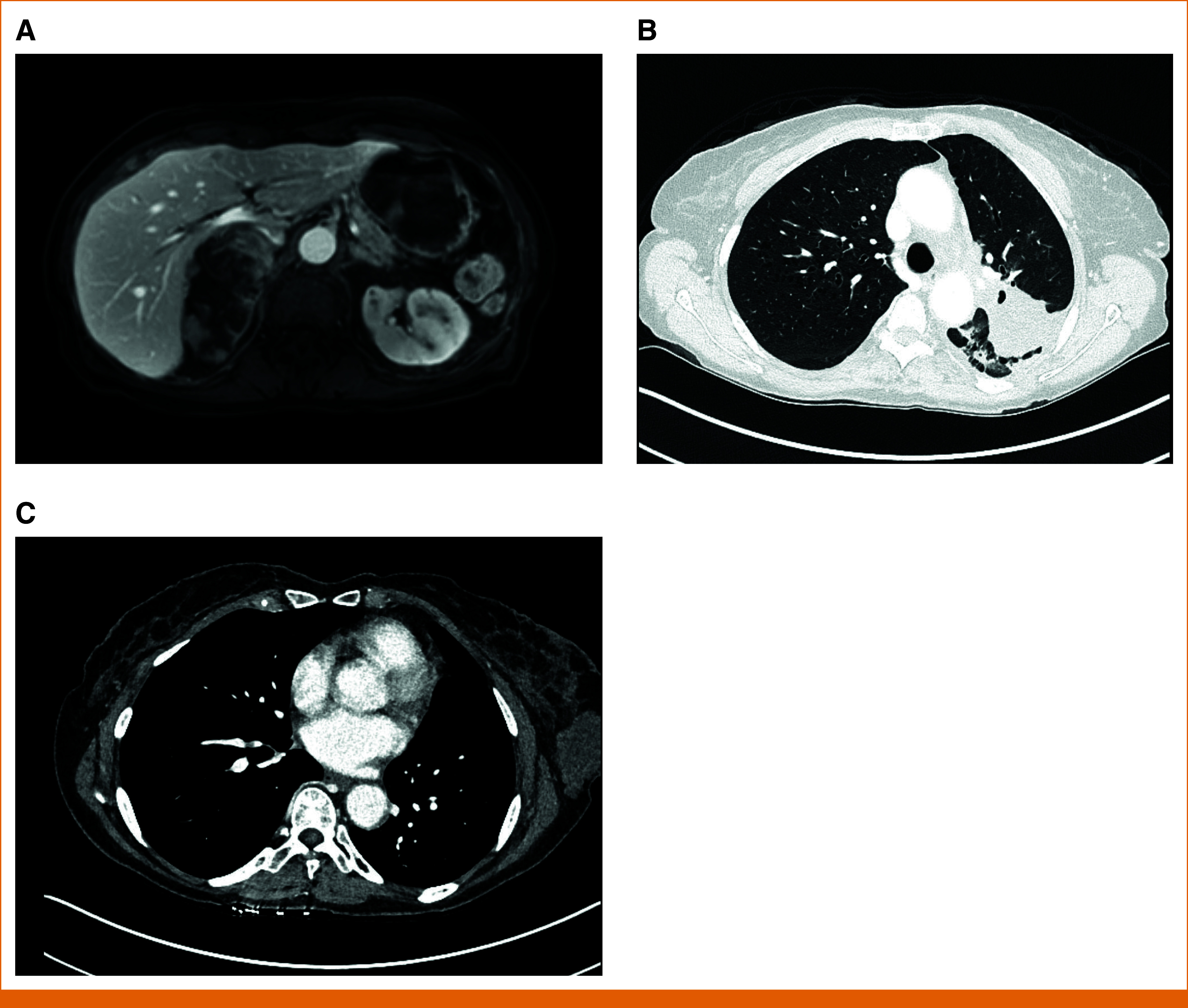
(A) Axial image from abdominal magnetic resonance imaging performed 6 months after treatment initiation shows a right adrenal mass with marked restricted diffusion and progressive hypoenhancement, measuring 8.5 × 5.1 cm. Mass effect on the adjacent right hepatic lobe, IVC, and liver hilum, as well as mild inferior displacement of the right kidney, is present. (B) Axial image from chest CT with contrast performed 6 months after treatment initiation shows the continuous decrease in size of the left upper lobe mass to 4.8 cm in largest dimension. (C) Axial image from chest CT with contrast performed 6 months after treatment initiation shows the continuous decrease in size of left breast mass to 51.9 mm in largest dimension. CT, computed tomography; IVC, inferior vena cava.

Six months after treatment initiation, she developed low back pain. Abdominal CT was suspicious for disease progression involving the right adrenal gland, later confirmed on fine-needle aspiration with IHC positive for keratin and TTF-1; all other sites of disease continued to demonstrate treatment response (Fig 4). PB NGS was obtained, and MET p.Y1230C (NM_000245.4(MET):c.3689A>G (p.Tyr1230Cys)) and MET p.Y1230H (NM_000245.4(MET):c.3688T>C (p.Tyr1230His)) mutations were identified in addition to the known MET exon 14 skipping mutation. Tissue NGS also identified the MET p.Y1230H mutation (Table 1). Surgical resection of the sole enlarging metastasis was pursued, but the procedure was aborted intraoperatively because of interval growth of the adrenal mass such that it was inseparable from the inferior vena cava. The patient was referred for palliative radiation therapy to the symptomatic adrenal metastasis. Currently, second-line systemic therapy with carboplatin, pemetrexed, and pembrolizumab is being considered.

## Statement of Informed Consent

Written informed consent was obtained from the patient for publication of this case report and accompanying images.

## Discussion

Accurate cancer diagnosis can be challenging. The initial workup of this patient was consistent with triple-negative MBC, a rare, aggressive, and heterogeneous malignancy with poor prognosis that comprises epithelial and sarcoma-like mesenchymal cells. Patients with advanced-stage disease generally require surgical resection, radiation, and systemic therapy with chemotherapy and immunotherapy, which can be associated with significant treatment-related toxicity.^2^ Interestingly, further investigation revealed a mass in the lung, leading to a revised diagnosis of lung adenocarcinoma with metastasis to the breast, an unusual finding. MNSCLC commonly spreads to the pleura, brain, adrenal glands, lungs, liver, and bones.^3^ In an investigation of 1,549 patients with mNSCLC, 10 additional metastatic sites were identified, none of which included the breast.^3^

Our patient was found to have activation of the MET proto-oncogene by exon 14 skipping, which has a median frequency of 2.4% in NSCLC (adenocarcinoma) and historically has been associated with poor prognosis.^4^ While MET exon 14 skipping has been seen in other cancer types, including gastric, colorectal, and CNS, only in NSCLC has the efficacy of MET TKIs been well established. In 2020, the MET TKI capmatinib was approved by the US Food and Drug Administration (FDA) in any treatment line for MET exon 14 skipping–mutated NSCLC.^4^ A review of capmatinib use in the first line revealed a median overall response rate of 68.8%, a median progression-free survival of 10.6-12.5 months, and an overall survival of approximately 20.8 months.^4^ The most common adverse drug reactions associated with capmatinib are peripheral edema, nausea, and vomiting.^5^ If our patient's diagnosis had not changed, then the side effects from MBC treatment with chemotherapy, with or without immunotherapy, would have likely been worse as compared with her excellent tolerance of capmatinib.

PB and tissue NGS was obtained on identification of disease progression in the right adrenal gland and revealed interval development of MET p.Y1230C and MET p.Y1230H mutations. In vitro models have identified MET Y1230 as a hotspot for secondary resistance mutations in patients with NSCLC who have been treated with type I MET TKIs like capmatinib.^6^ Type I TKIs bind to MET's active form, whereas Type II MET TKIs bind to MET's inactive form. Using in vitro models, Fujino et al^6^ demonstrated that NSCLC with resistance mutations to type I TKIs generally responds to Type II TKIs like cabozantinib.

Clinically, this phenomenon was observed in a patient whose lung cancer carried both epidermal growth factor receptor (EGFR) and MET mutations.^7^ This patient was treated concomitantly with EGFR TKI osimertinib and type I MET TKI savolitinib. After 18 months, the patient acquired resistance mutations, including one shared by our patient: MET p.Y1230C. Savolitinib was replaced with type II TKI cabozantinib, and his disease stabilized. Repeat PB NGS obtained months later revealed the elimination of the MET p.Y1230C mutation, suggesting treatment of the associated type I TKI-resistant clone. Type II MET TKIs are the subject of multiple ongoing clinical trials for MET-amplified NSCLC, but they are not yet approved by the FDA. Compassionate use of cabozantinib has been discussed with and declined by our patient.

This case highlights the effective use of NGS, which identified an actionable mutation seen in lung adenocarcinoma that, in addition to extended IHC evaluation, supported the revision of the patient's diagnosis to NSCLC. Although National Comprehensive Cancer Network (NCCN) guidelines do not advise relying on NGS alone to identify cancer of unknown primary, NGS results may spur additional investigation; furthermore, NCCN does not discourage supplemental use of NGS for therapeutic purposes. Finally, this case illustrates the utility of PB NGS, that is, circulating tumor DNA, to monitor disease response and detect emergent subclonal mutations or resistant clones.^8^

The primary strength of this case's management was the BC team's broad differential in the face of uncertain histopathology and pursuit of further clinical investigation to confirm a diagnosis. Breast metastasis is an uncommon presentation of NSCLC, but it should be considered in cases like that of this patient.

In conclusion, in the absence of heightened suspicion for a nonbreast primary tumor, our patient might have been misdiagnosed with triple-negative MBC, potentially exposing her to increased treatment-related toxicities from cytotoxic chemotherapy and poor MBC-specific prognosis without survival benefit from targeted therapy for NSCLC. As this unusual case shows, lung cancer should be considered in the differential diagnosis of MBC and undifferentiated breast malignancy. NGS can be used effectively in cases of undifferentiated malignancy for diagnostic and therapeutic purposes, for both initial therapy and detection of resistance mutations that could guide second-line treatment.
